# Androgen deprivation therapy in castrate-resistant prostate cancer: how important is GnRH agonist backbone therapy?

**DOI:** 10.1007/s00345-014-1406-2

**Published:** 2014-09-27

**Authors:** Axel S. Merseburger, Peter Hammerer, Francois Rozet, Thierry Roumeguère, Orazio Caffo, Fernando Calais da Silva, Antonio Alcaraz

**Affiliations:** 1 Department of Urology and Urologic Oncology, Hannover Medical School, Hannover, Germany; 2 Department of Urology, Academic Hospital Braunschweig, Brunswick, Germany; 3 Department of Urology, L’Institut Mutualiste Montsouris, Paris, France; 4 Department of Urology, Hôpital Erasme, Université Libre de Bruxelles, Brussels, Belgium; 5 Department of Medical Oncology, Santa Chiara Hospital, Trento, Italy; 6 Department of Urology, Centro Hospitalar Lisboa Central, Lisbon, Portugal; 7 Department of Urology, Hospital Clínic Universitat de Barcelona, Barcelona, Spain

**Keywords:** Castrate-resistant prostate cancer, Individualised management, Backbone androgen deprivation therapy

## Abstract

**Background:**

A growing number of treatment options exist to treat metastatic castrate-resistant prostate cancer (mCRPC), and with these newer options, many questions about optimising treatment remain unanswered. One recommendation that may potentially be overlooked by practitioners is that androgen deprivation therapy (ADT) should be maintained when CRPC develops and when treatment with any of the newer agents is initiated.

**Aim:**

However, to emphasise this recommendation, it is valuable to interrogate the evidence for maintaining ADT in different clinical situations.

**Outcome:**

This statement, reflecting the views of the authors, provides a discussion of this evidence and the rationale behind the recommendation that ADT should be continued in CRPC.

## Introduction

The European Association of Urology (EAU) guideline clearly states that when castrate-resistant prostate cancer (CRPC) develops, androgen deprivation therapy (ADT) should be continued indefinitely; this recommendation applies to metastatic CRPC (mCRPC) and non-metastatic CRPC (nmCRPC) [1]. Other guidance, such as that from the American Urological Association (AUA) [2] and the National Comprehensive Cancer Network (NCCN) [3], likewise mention the need to maintain ADT when CRPC develops. Gonadotropin-releasing hormone (GnRH) agonist treatment is considered the mainstay of ADT, and should therefore be continued when men receiving ADT develop CRPC.

However, there appears to be a risk that the need to continue ADT may be overlooked by some practitioners. This aspect of the guidelines is sometimes not mentioned in subsequent discussions. Furthermore, although randomised controlled trials of the many newer agents for mCRPC, including abiraterone, enzalutamide, sipuleucel-T, radium 223 and cabazitaxel [4–10], all had continuation of ADT and mainstay of castration levels of testosterone (<50 ng/dl) as an inclusion criterion, the fact that these agents are being used in combination with ADT is not emphasised.

Lowering testosterone levels is associated with improved survival in prostate cancer [11], and this underlines the need to maintain androgen deprivation in patients. This often-quoted study showed that survival was significantly greater if serum testosterone levels were <20 ng/dl than if they were 20–50 ng/dl, and survival was worst if serum testosterone was >50 ng/dl—however, only 73 patients with non-metastatic prostate cancer were included in this analysis [11]. In metastatic disease, while hormonal treatment improves symptoms, there is no conclusive prospective evidence that lowering testosterone levels improves life expectancy [12].

Likewise, when chemotherapy is initiated in CRPC patients, the EAU recommends continuing the backbone ADT [1]. This guideline is based upon a single study of androgen priming in a small group of patients (*n* = 85) and using chemotherapy regimens that are now outdated [13]. Therefore, this recommendation is also worthy of scrutiny.

However, it is becoming clear that within the prostate and prostate tumour microenvironment androgen activity continues even when serum testosterone levels are suppressed by ADT [14], and intracrine androgen synthesis is sufficient to activate androgen receptor target genes [15]. Adaptive alterations include alternative androgen synthesis pathways, and androgen receptor overexpression, mutation and splice variations [16]. Furthermore, many mechanisms that may confer castration resistance still require, or are enhanced by, the presence of androgens or androgen receptor ligands. Together these observations suggest that treatment combinations that include ADT and suppress intracrine and systemic androgen contributions are required in CRPC.

In December 2013, a group of experts (the authors of this paper) met to explore the evidence and rationale for continuing ADT in CRPC when other treatments are initiated. The aim is to provide clear statements on this issue in this manuscript. Before the meeting, participants were assigned to specific topics and conducted PUBMED searches on the recent literature on these topics. Assigned participants presented on these topics during the meeting, and all participants then developed the recommendations and contents of this paper based on all the reports presented at the meeting. As such, the contents of this paper represent the conclusions of the authors only. References in the text have been assessed according to their level of scientific evidence (Table 1), and recommendations have been graded according to the Oxford Centre for Evidence-based Medicine Levels of Evidence as used in the EAU guidelines (Table 2) [1, 17].

**Table 1:** Level of evidence

Level	Type of evidence
1a	Evidence obtained from meta-analysis of randomised trials
1b	Evidence obtained from at least one randomised trial
2a	Evidence obtained from one well-designed controlled study without randomisation
2b	Evidence obtained from at least one other type of well-designed quasi-experimental study
3	Evidence obtained from well-designed non-experimental studies, such as comparative studies, correlation studies and case reports
4	Evidence obtained from expert committee reports or opinions or clinical experience of respected authorities

**Table 2:** Grade of recommendation

Grade	Nature of recommendations
A	Based on clinical studies of good quality and consistency that addressed the specific recommendations, including at least one randomised trial
B	Based on well-conducted clinical studies, but without randomised clinical trials
C	Made despite the absence of directly applicable clinical studies of good quality

## The rationale for ADT use with abiraterone

Abiraterone selectively inhibits the enzyme 17 α-hydroxylase/C17, 20-lyase (CYP17) and thus inhibits androgen biosynthesis [18]. In CRPC, abiraterone acetate has been shown to achieve sustained suppression of testosterone in blood and bone marrow aspirate to <pg/ml levels, when added to continuing backbone ADT [19]. Abiraterone also has direct activity on reducing the expression of the androgen receptor gene [20]. Therefore, the need to eliminate as many parts of the androgen receptor signalling pathway as possible provides a rationale for combining abiraterone with ADT.

Crucially, experimental evidence suggests that the testosterone suppression achieved by abiraterone monotherapy is not sustained in non-castrated men and is overcome by a subsequent twofold–threefold surge in luteinising hormone (LH) levels [21] [level of evidence (LoE): 2b]. Conversely, the addition of abiraterone to backbone ADT results in sustained decreases in testosterone and adrenal steroid concentrations [22, 23]. Although the pharmacokinetic study of O’Donnell et al. [21] assessed a small number of men, it does suggest a need to maintain castrate levels of testosterone with ADT when initiating abiraterone therapy.

This rationale has been used in phase III trials of abiraterone. The efficacy of abiraterone (plus prednisolone) was demonstrated in two pivotal trials in patients with mCRPC; in one study, abiraterone was used before chemotherapy, and in one study, it was used after chemotherapy [4, 5] (LoE: 1b). Importantly, castration levels of testosterone were maintained in both these studies with the continuation of ADT.

**Fig. 1:**
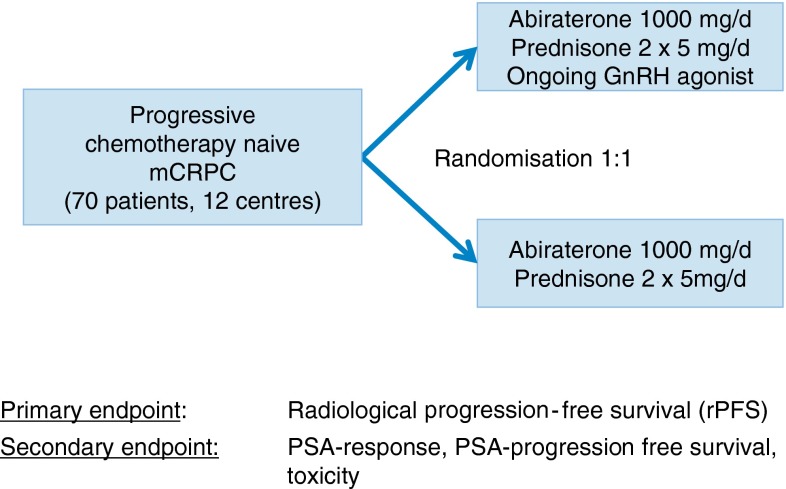
Design of the ongoing SPARE trial

To date, however, there have been no clinical trials comparing abiraterone (plus prednisolone) monotherapy with abiraterone plus ADT to confirm the need for continued ADT when initiating abiraterone therapy in patients with mCRPC. The planned German multicentre trial SPARE will investigate the impact of continuing ADT when initiating abiraterone. This study (German Association of Urological Oncology trial number AUO 67/11) will investigate abiraterone monotherapy (plus prednisolone) versus abiraterone plus ADT (plus prednisolone) in 70 men with chemotherapy-naïve mCRPC (Fig. 1). Preliminary results of this study may be available in 2016, and these are eagerly awaited as they will provide the first prospective insight on the potential efficacy advantages of maintaining ADT when abiraterone treatment is initiated in mCRPC.

Another concept that could be considered is whether abiraterone could be used before CRPC develops, either as a monotherapy alternative to ADT or in combination with ADT. A number of ongoing trials are investigating this (Table 3). Data are available from one of these trials comparing abiraterone + prednisone + leuprolide with leuprolide alone in men with localised high-risk prostate cancer, and these suggest that testosterone suppression within prostate tissue is superior with the combination treatment but dihydrotestosterone levels in prostate tissue are lower with ADT alone. Prostate-specific antigen (PSA) response was significantly higher in the combination treatment arm after 12 weeks. Such studies on earlier treatment with ADT and abiraterone combination may also provide important information on the potential risk that earlier and more aggressive treatment could accelerate the time to CRPC development (or increase metabolic side effects). Patient selection (of those with more aggressive disease) will be important for the future of such aggressive treatment regimens, but better prognostic markers may be needed to make such patient selection possible.

**Table 3:** Ongoing studies investigating abiraterone combined with backbone ADT

ClinicalTrials.gov identifier	Overview of design	Patients	Expected results
NCT00924469	Abiraterone + prednisone + leuprolide versus leuprolide alone for 12 weeks	58 men with localised high-risk prostate cancer	Available at: http://clinicaltrials.gov/ct2/show/results/NCT00924469?sect=X6015#outcome1
NCT01786265	Abiraterone + prednisone + leuprolide versus leuprolide alone for 12 months	Approximately 200 men with PSA progression after radical prostatectomy (RP) and/or radiation therapy	2017
NCT01715285	ADT versus ADT + abiraterone (+prednisolone)	Approximately 1,270 men with high-risk metastatic hormone-naïve prostate cancer	2018
NCT00268476 (STAMPEDE) [45]	Several treatment arms (all with a backbone of ADT) including one with abiraterone added to ADT	Approximately 5,000 men with hormone-naïve advancing or metastatic prostate cancer	2017
NCT01946165	GnRH agonist + abiraterone (+prednisolone) + enzalutamide versus GnRH agonist + abiraterone (+prednisolone)	Approximately 66 men in the pre-operative setting with prostate cancer with a high risk of recurrence	2021
NCT01751451	Abiraterone versus degarelix versus abiraterone + degarelix	Approximately 120 men with PSA progression after RP (with or without nodal disease)	2014

Of all the ongoing studies of abiraterone with or without ADT, clearly the SPARE study will provide some valuable insight into the need to maintain a backbone of ADT when initiating abiraterone therapy in mCRPC. Until these data are available, and with all phase III trial data of abiraterone having ongoing ADT as an essential inclusion criterion, ADT plus abiraterone may be considered the standard of care in many men with asymptomatic or mildly symptomatic mCRPC (Grade B recommendation).

Interestingly, there are no published data assessing abiraterone (with or without ADT) in patients with non-metastatic CRPC (nmCRPC). More data on the use of newer agents for the treatment of nmCRPC are needed.

## The rationale for ADT use with enzalutamide

Androgen receptor signalling persists during castration, and several mechanisms, even in individual patients (through clonal heterogeneity), may explain this persistence [24]. Addition of androgen receptor blockers to ADT may therefore help achieve more complete androgen blockade.

The androgen receptor blocker bicalutamide has been used for many years in combination with ADT to achieve complete androgen blockade. However, in advanced metastatic prostate cancer, monotherapy with bicalutamide was inferior to ADT in prolonging overall survival (OS) [25] (LoE: 1b). Bicalutamide monotherapy also resulted in a high frequency of gynaecomastia (approximately 70 % of patients) [26], but this frequency reduced when combined with ADT (LoE: 1b). Bicalutamide may also function as an androgen receptor agonist when androgen receptors are overexpressed (which may occur in up to 30 % of CRPC tumours) [27–29], with certain mutations of the androgen receptor [30] or in the setting of inflammation [31, 32].

Enzalutamide is a novel androgen receptor blocker that inhibits nuclear translocation of androgen receptors by localising the nuclear N-terminal of the androgen receptor to the cytoplasm [33]. Enzalutamide binds to the androgen receptor with eightfold higher affinity than bicalutamide [34]. With the development of enzalutamide, it is important to ask whether there is any clinical difference between enzalutamide and bicalutamide and whether this influences the need to combine enzalutamide treatment with ADT.

Unlike bicalutamide, enzalutamide has no known agonist activity, and it is thought that bicalutamide resistance does not exclude subsequent enzalutamide use [34]. In addition, a recent report has assessed enzalutamide monotherapy in hormone-naive men with prostate cancer [35]. This initial report of enzalutamide monotherapy in 67 patients suggested a lower frequency of gynaecomastia (36 %) than previously reported with bicalutamide monotherapy. Furthermore, PSA declines were of a similar magnitude to those achieved by ADT but adverse events were frequent and testosterone levels increased [35] (LoE: 2a). Therefore, more data are needed to determine whether the combination of enzalutamide with ADT has a favourable efficacy and safety profile for the treatment of CRPC compared with enzalutamide monotherapy.

In the meantime, as with abiraterone, pivotal trials of enzalutamide in men with CRPC included the need for castration maintenance with ADT [6, 7], and these studies have shown that this combination improved OS when used before chemotherapy and after chemotherapy (LoE: 1b). Furthermore, there was a low frequency of seizure as a side effect of enzalutamide [7] suggesting that the safety profile of enzalutamide does not represent a problem when combining it with ADT (LoE: 1b).

Ongoing clinical studies may provide some clues to the need for backbone ADT when initiating enzalutamide in patients with CRPC (Table 4). However, there are no trials with enzalutamide that are equivalent to the SPARE study with abiraterone, which directly compare enzalutamide monotherapy with enzalutamide combined with ADT in men with CRPC. In the absence of any such data or the prospect of such data being published, the potential adverse event of gynaecomastia with enzalutamide monotherapy, and with all phase III trial data of enzalutamide having ongoing ADT as an essential inclusion criterion, backbone ADT should be continued when initiating enzalutamide. Potential long-term safety considerations of combining ADT with enzalutamide need to be assessed further.

**Table 4:** Ongoing studies investigating enzalutamide and ADT

Trial identifier	Overview of design	Patients	Expected results
NCRN322 (TERRAIN)	ADT + enzalutamide versus ADT + bicalutamide	370 men with mCRPC	December 2014
NCT01547299	Enzalutamide monotherapy versus enzalutamide + leuprolide + dutasteride	As neoadjuvant treatment in approximately 50 men with localised prostate cancer who are undergoing RP	Trial completed in 2013
NCT02003924 (PROSPER)	ADT + enzalutamide versus ADT alone	Approximately 1,560 men with nmCRPC	2017

## ADT during chemotherapy

EAU guidelines state that ADT with GnRH analogues should be continued when giving mCRPC patients chemotherapy [1]. This is based upon the data of a single study [13], in which 85 men with CRPC refractory to orchiectomy receiving a now outdated chemotherapy regimen (and thus, the survival time was shorter than would be expected today) had worse median survival if they were androgen primed (10 vs. 15 months if not primed) (LoE: 3).

The rationale for continuing ADT when starting chemotherapy in mCRPC is that stopping ADT may lead to renewed release of testosterone and stimulation of the remaining androgen-sensitive elements of the tumour. Conversely, approximately 50 % of men receiving ADT in the long-term remain castrated for 2.5 years after stopping ADT [36] (LoE: 3), and stopping ADT may re-induce hormone sensitivity [37] (LoE: 2b). These conflicting viewpoints are difficult to prove as there is a lack of well-designed prospective trials exploring this issue, and retrospective data are conflicting [38–40]. There is no strong evidence that the combination of ADT with chemotherapy causes harm but there is also no strong evidence of benefit.

Recent pivotal trials of chemotherapy in prostate cancer have stipulated that ADT should be continued when chemotherapy was initiated [10, 41] (LoE: 1b), and guidelines all recommend continuation of ADT when initiating chemotherapy [1, 42]. Furthermore, the ChemoHormonal therapy versus Androgen Ablation Randomized Trial for Extensive Disease in prostate cancer (CHAARTED; ClinicalTrials.gov identifiers: NCT00309985) recently reported that in hormone-naïve men with metastatic prostate cancer, OS was improved and metastatic load was decreased when treatment was initiated with ADT plus chemotherapy versus ADT alone (http://www.nih.gov/news/health/dec2013/nci-05.htm). Conversely, an open-label phase III trial showed no benefit of adding chemotherapy to ADT as first-line treatment compared with ADT alone in hormone-sensitive men with metastatic prostate cancer [43] (LoE: 1b). These conflicting results do not help clarify the value of maintaining ADT when initiating chemotherapy in men with mCRPC, and care should be taken if trying to extrapolate these data to the mCRPC setting.

An alternative approach to that currently recommended may be to stop ADT when starting chemotherapy and then monitor testosterone levels; restarting ADT when testosterone levels go above the threshold for castration. Two ongoing studies (ClinicalTrials.gov identifiers: NCT01487902 and NCT01224405) are investigating the possible advantage of maintaining ADT during chemotherapy. One of these studies (NCT01487902) that is comparing docetaxel plus prednisolone with docetaxel plus prednisolone and leuprolide in approximately 90 men with CRPC was due for completion in October 2013, and results may therefore be available soon. Until these results are available ADT should be continued when chemotherapy is initiated in mCRPC in daily practice.

## Discussion

All clinical trials of newer agents (and recent trials of chemotherapy agents) in mCRPC include patients who maintain castrate levels of testosterone, and so clinical practice should adhere to this principle of continuing ADT when initiating abiraterone, enzalutamide or chemotherapy. Likewise, experimental agents that are in advanced stages of clinical development for treatment of CRPC are all being assessed in patients who maintain their castration status with ADT, and so this recommendation is likely to apply to other agents that may be registered in the next few years. However, not only are more prospective data needed to assess the importance of backbone ADT in CRPC, but also reliable prognostic and predictive biomarkers are urgently needed to individualise treatment with newer agents, their combination with ADT, and the optimum treatment sequences.

Another consideration is that ADT as the standard of care may be moving earlier in the disease continuum, and the availability of newer agents means that optimum treatment sequences that include ADT need to be ascertained in CRPC and in less advanced prostate cancer.

Finally, more trial data are urgently needed on the management of patients with nmCRPC; EAU definition of CRPC does not require the presence of metastases but it is possible that all patients with CRPC have metastatic disease and those classified as having nmCRPC have metastases that cannot be detected with current techniques. Irrespective of this, very few studies have included appreciable numbers of patients that would currently be considered nmCRPC. In practice, the lack of data means that maintaining ADT in these nmCRPC patients is the recommended treatment option—it is unclear if bone-targeted treatment is also useful in this setting. For example, in the Zometa European Study (ZEUS), zoledronic acid did not prevent bone metastases or improve OS in M0 patients (http://www.medscape.com/viewarticle/781457). Whereas, the 147 trial suggested that denosumab may delay the onset of skeletal-related events (which included bone metastases) in patients with CRPC, but the adverse event profile was not favourable [44] (LoE: 1b).

## Panel recommendations

- The emergence of several agents for the management of CRPC has the potential to prolong and enhance the life of men with this disease. As data emerge on these new agents and on improving biomarkers, a more individualised approach to the use of these newer agents and their combination with ADT will optimise management further (Grade C recommendation).
- As all randomised prospective data for abiraterone use in CRPC include the continuation of backbone ADT, and based upon the findings of O’Donnell et al. [21] that suggested abiraterone monotherapy does not sustain testosterone suppression in non-castrated men, ADT should be maintained in men with CRPC when they initiate abiraterone treatment (Grade B recommendation).
- All phase III trial data of enzalutamide in CRPC include the continuation of backbone ADT. This combined with the potential adverse event of gynaecomastia with enzalutamide monotherapy suggest that ADT should be continued when initiating enzalutamide in men with CRPC (Grade B recommendation).
- When initiating chemotherapy in CRPC, data on the benefits of continuing ADT are conflicting. However, in the absence of any clear evidence of harm and because prospective phase III trial data maintain ADT, it is recommended that backbone ADT is maintained when chemotherapy is initiated in CRPC (Grade B recommendation).
